# Notching and Pulsatility Index of the Uterine Arteries and Preeclampsia in Twin Pregnancies

**DOI:** 10.3390/jcm9082653

**Published:** 2020-08-15

**Authors:** Stephanie Springer, Mariella Polterauer, Maria Stammler-Safar, Harald Zeisler, Heinz Leipold, Christof Worda, Katharina Worda

**Affiliations:** 1 Department of Obstetrics and Gynecology, Medical University of Vienna, 1090 Vienna, Austria; stephanie.springer@meduniwien.ac.at (S.S.); mariella.polterauer@meduniwien.ac.at (M.P.); maria.stammler-safar@meduniwien.ac.at (M.S.-S.); harald.zeisler@meduniwien.ac.at (H.Z.); katharina.worda@meduniwien.ac.at (K.W.); 2 Department of Obstetrics and Gynecology, Landeskrankenhaus Klagenfurt, 9020 Klagenfurt am Wörthersee, Austria; heinz.leipold@lku-klu.at

**Keywords:** uterine arteries, notching, pulsatility index, preeclampsia, twin pregnancy

## Abstract

Increased uterine artery Doppler indices have been shown to be associated with preeclampsia and adverse pregnancy outcomes in singleton and twin pregnancies. At 20–22 weeks of gestation, we assessed the use of notching, the highest, lowest, and mean pulsatility index (PI), and the combination of notching and PI of the uterine arteries to screen for preeclampsia. This was done in a cohort of 380 twin pregnancies. The results showed that the combination of notching and the highest PI above the 95th centile of the uterine arteries gives the best screening characteristics for preeclampsia in twin pregnancies. We calculated sensitivities for preeclampsia for notching, highest PI, and the combination of notching and the highest PI of 50%, 45% and 91%, with specificities of 96%, 96% and 93%, respectively. The present findings demonstrate that notching, increased highest PI, and the combination of notching and the highest PI of the uterine arteries is associated with an increased risk of preeclampsia in twin pregnancies. We observed the highest sensitivity and specificity by using the combination of notching and the highest PI of the uterine arteries.

## 1. Introduction

Twin pregnancies are associated with higher risks of mortality and morbidity than singleton pregnancies, due to more maternal and fetal complications during pregnancy and a higher rate of preterm deliveries [1]. Preeclampsia is a major cause of maternal and perinatal mortality and morbidity, and early detection to provide optimal surveillance and therapy is one of the most important goals in obstetrics [2,3,4].

Preeclampsia affects about 2% of singleton pregnancies, and 4–9% of twin pregnancies [5,6,7]. Advanced maternal age, larger placenta, artificial reproduction technologies, and higher rates of obesity might contribute to the higher incidence of preeclampsia in twin pregnancies [2,8]. In pregnancies with preeclampsia, trophoblastic invasion of the spiral arteries is impaired and placental resistance is high [9]. Doppler ultrasound can be used to assess blood flow velocity in the uterine arteries and thus potentially identify women at increased risk for preeclampsia [10,11,12]. Increased uterine artery Doppler indices have been shown to be associated with preeclampsia and adverse pregnancy outcomes in singleton and twin pregnancies, although sensitivity has been lower in twin pregnancies [13,14,15].

In a previous study, we showed that the higher Pulsatility Index (PI) of the two uterine arteries above the 95th centile is a significant risk factor for preeclampsia and adverse pregnancy outcomes in twin pregnancies, with a sensitivity and specificity of 27% and 98%, respectively [16]. The actual aim of the study was to assess the additional use of notching in combination with the mean, lowest, and highest PI of the uterine arteries, in order to screen for preeclampsia in twin pregnancies.

## 2. Materials and Methods

In this screening study, we investigated the use of notching, the mean, lowest, and highest pulsatility index (PI) of the uterine arteries to assess the risk for preeclampsia and adverse pregnancy outcomes associated with uteroplacental insufficiency in twin pregnancies. The study was performed according to the standards of the Helsinki Declaration and was approved by the Institutional Review Board of the Medical University of Vienna (IRB number: 1512/2014). Informed consent was obtained from all participants.

### 2.1. Patients

Over a period of 3 years (2015–2018), 423 women with twin pregnancies who had a first trimester screening at 11+0 to 13+6 weeks of gestation, including determination of gestational age and chorionicity, and who received an anomaly scan including a Doppler assessment of the uterine arteries at 20–22 weeks of gestation, were included in the study. We excluded 45 patients (10.6%): 12 (2.8%) did not receive a validated first-trimester ultrasound scan; in 9 pregnancies (2.1%), fetal demise before 24 weeks occurred; 14 (3.3%) underwent selective feticide or reduction of multifetal pregnancies; in 6 pregnancies (1.4%), major fetal anomalies or chromosomal abnormalities were detected; and in 4 cases (0.9%), uterine artery PI was not measured at 20–22 weeks. Furthermore, 13 twin pregnancies (3.1%) were excluded because of missing outcome data. Therefore 380 women were eligible for the study.

### 2.2. Data Collection

The patients’ characteristics and medical history were recorded, gestational age was confirmed by measuring the crown-rump length of the larger twin at the first-trimester screening, and chorionicity was ascertained depending on the absence or presence of placental tissue extended into the base of the intertwin membrane before 14 weeks of gestation. In monochorionic twin pregnancies, no placental tissue was illustrated (T sign), and in dichorionic twin pregnancies, placental tissue was visible with an ultrasound (lambda sign). All women had an ultrasound examination to screen for fetal anomalies at 20–22 weeks of gestation. At this scan, notching and the PI of both uterine arteries were measured by transabdominal ultrasound. After identifying the uterine arteries (color flow mapping), a pulsed wave Doppler was conducted at the crossover of the uterine and external iliac arteries. When three similar consecutive waveforms were provided, the presence of notching was determined, and the PI was measured on each side. The lowest PI was defined as the lower measurement of both sides, and the highest PI was defined as the higher measurement of both sides. The Doppler waveforms were analyzed by two independent experienced sonographers, and only if both agreed, then notching was used for calculation. It has been shown that a quality control process can improve the quality of mean uterine artery pulsatility index measurements [17]. Therefore, only well-trained operators who followed the above-established protocol performed the uterine artery measurements. Each of them did at least 50 measurements before being accepted for this trial.

Routine clinical management after the anomaly scan and until delivery included visits every two weeks (monochorionic twins) and every four weeks (dichorionic twins), respectively. Each visit included the measuring of maternal blood pressure, performing a urine dipstick, fetal growth assessment, and measuring of the deepest pocket of the amniotic fluid of each fetus separately. Furthermore, fetal Doppler values of the umbilical artery and, if necessary, fetal Doppler values of the middle cerebral artery and the ductus venosus were assessed. Demographic characteristics and data on fetal and maternal outcomes were collected from the hospital maternity records.

### 2.3. Outcome Measures

The main outcome measure was preeclampsia. Preeclampsia was diagnosed according to the adapted criteria of the American College of Obstetrics and Gynecology: if systolic blood pressure was ≥140 mmHg or diastolic blood pressure was ≥90 mmHg on two occasions at least 4 h apart, developing after 20 weeks of gestation in previously normotensive women, and proteinuria was ≥ 0.3 g in a 24-hour urine specimen, or two readings of at least 2+ (>100 mg/dL) protein on a urine dipstick if quantitative methods were not available. In women with new-onset hypertension, the new onset of any of the following was diagnostic of preeclampsia, even if proteinuria was not present: a platelet count of <100,000/microL, serum creatinine of >1.1mg/dL, liver transaminases at least twice the normal concentrations, pulmonary edema, or cerebral or visual symptoms [18]. Secondary outcome measures were early preeclampsia (<34 weeks of gestation) and fetal growth restriction (FGR) below the 10th centile. Special growth charts for twins were used [19].

The relationship between notching, elevated mean, lowest, and highest PI, and preeclampsia as well as early preeclampsia and fetal growth restriction was evaluated. 

Sensitivity, false-positive rate, positive predictive value and negative predictive value with 95% confidence intervals for notching, a cutoff mean, lowest and highest PI above the 95th centile and for the combination of notching and the highest PI above the 95th centile for preeclampsia, early preeclampsia and fetal growth restriction were calculated. 

### 2.4. Statistical Methods

SPSS software (version 23.0; SPSS, Chicago, IL, USA) was used for statistical analyses. Parametric continuous variables are summarized as means (± SD), non-normally distributed continuous variables as medians (minimum and maximum), and categorical data as percentages. A Kolmogorov–Smirnov test was used to identify non-normally distributed continuous variables. Categorical variables were analyzed with a chi square test or Fisher’s exact test, continuous variables were compared with unpaired Student’s *t*-test, Kruskal–Wallis, and Mann–Whitney U test with post hoc Bonferroni correction for multiple testing (critical statistical significance *p* < 0.03). Detection rates of all pregnancies complicated by preeclampsia were calculated. Receiver-operating characteristics (ROC) curves were constructed and the area under the curve was calculated (*p* < 0.05 was considered significant).

## 3. Results

Of 380 twin pregnancies that were analyzed, 27.1% (103/380) were monochorionic and 72.9% (277/380) were dichorionic twin pregnancies. Of the total number of pregnancies, 24 women (6.3%) developed preeclampsia, 15 women (3.9%) developed early preeclampsia, and in 78 (20.5%) FGR was diagnosed. 

Patient characteristics are shown in Table 1. Patients with preeclampsia were significantly older, had a higher body mass index, and more frequently underwent assisted reproduction than women without preeclampsia.

The 95th centile of the mean, lowest and highest PI of the uterine arteries was 1.31 (range 0.43–3.12), 1.14 (range 0.34–2.73) and 1.54 (range 0.48–3.5), respectively. Unilateral or bilateral notches were seen in 15 and 4 women, respectively. PI and notching did not significantly differ between monochorionic and dichorionic twins (mean PI 0.87 vs. 0.84, *p* = 0.28; lowest PI 0.76 vs. 0.73, *p* = 0.30; highest PI 0.98 vs. 0.95, *p* = 0.26; notching 9.6 vs. 7.2%; *p* = 0.67).

We investigated the relationship between notching of the uterine arteries and preeclampsia. Furthermore, we investigated the use of the mean, lowest and highest PI, as well as maternal characteristics for the prediction of preeclampsia. Each single parameter was significantly associated with preeclampsia (Table 2). The combination of notching and the highest PI, together with maternal characteristics, had the highest impact in predicting the risk for preeclampsia. Of the 24 cases of preeclampsia: 11 women had notching, 10 had highest PI above the 95th centile and one woman had both notching and highest PI when measuring the uterine arteries at 20–22 weeks of gestation.

The relative risk (RR) for fetal growth restriction (FGR) < 10th centile using the highest, lowest, and mean PI and notching was 3.7 (1.6–8.5; *p* < 0.001), 3.2 (1.2–8.9; *p* < 0.01), 4.3 (1.6–11.1; *p* < 0.001), 4.1 (1.8–9.1; *p* < 0.01), respectively. The RR for FGR < 10th centile using the combination of notching and the highest PI was 3.5 (1.8–6.8, *p* < 0.01). We also analyzed left and right uterine artery traces but could not find any differences in predicting preeclampsia (Relative Risk (95% Confidence Interval) 4.8 (1.4–16.0) and 4.8 (1.7–13.3), respectively).

The screening characteristics for the development of preeclampsia, early preeclampsia, and FGR <10th centile for PI, notching and combination of these parameters are shown in Table 3.

**Figure 1:**
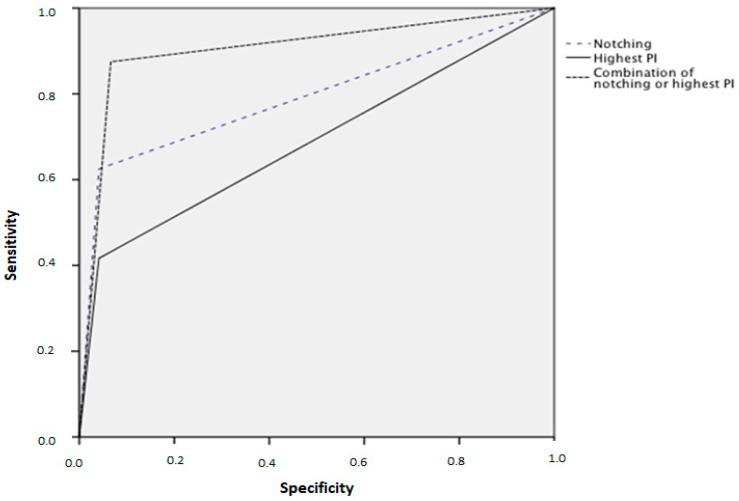
Screening receiver operating characteristics curves for prediction of preeclampsia.

**Figure 2:**
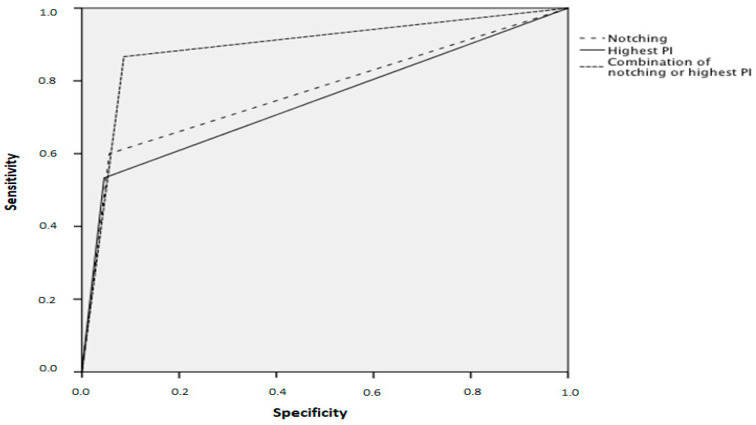
Screening receiver operating characteristics curves for prediction of early preeclampsia.

Receiver-operating characteristics curves for the prediction of preeclampsia and early preeclampsia are shown in Figure 1 and Figure 2. The best prediction for preeclampsia and early preeclampsia is calculated by the use of the combination of notching and the highest PI.

## 4. Discussion

In our study, we investigated whether notching increased the screening characteristics for preeclampsia, early preeclampsia, and FGR in twin pregnancies. The lowest, mean, and highest PI above the 95th centile as well as notching increased the risk for developing preeclampsia, early preeclampsia, and FGR in twin pregnancies. This was consistent with our previously published study, and including notching in the calculation further increased the prediction of preeclampsia. Notching showed, for preeclampsia, early preeclampsia, and FGR, higher sensitivity rates than all other parameters. When the highest PI was combined with notching, sensitivity rates of nearly 90% could be achieved. Therefore, the use of notching in a clinical setting could improve prediction rates of preeclampsia, early preeclampsia, and FGR. Typically, notching is seen in combination with an increased PI, but in some cases, notching was the only pathological finding in uterine artery Doppler ultrasound. 

Screening for preeclampsia in the first trimester can identify patients who are at high risk for developing preeclampsia. This effective screening includes a combination of mean arterial pressure, uterine artery Doppler, serum placental growth factor (PLGF), and pregnancy-associated plasma protein A (PAPP-A) together with maternal factors. In singleton pregnancies, detection rates of nearly 90% with a screen-positive rate of 10% could be achieved. This is true for Caucasian women—in women of Afro-Caribbean racial origin, the screen-positive rate increased to 34% with the same detection rate [20]. When using this model at the first trimester to predict preeclampsia in twin pregnancies, high detection rates up to 100% could be achieved, but at a high screen-positive rate of 75% [21]. This approach, with combining different maternal factors and biomarkers, has improved the false positive rate dramatically [22]. The ASPRE trial has shown in singleton pregnancies, which were screened in the first trimester and were at high risk for developing preeclampsia, that the administration of 150 mg aspirin can reduce the rate of early preeclampsia with delivery <32 weeks of gestation by about 90% [23]. The results with twin pregnancies are conflicting. There is a preliminary report which might show dose-dependent effects for the reduction in preeclampsia in twin pregnancies [24].

We have shown that the PI of the uterine arteries is lower in twin pregnancies than in singleton pregnancies, as reported earlier. There are three studies which investigated the associations between PI, the resistance index (RI), and notching of the uterine arteries and preeclampsia. Geipel et al. reported an association between elevated RI, notching, and preeclampsia only in dichorionic twins [15]. However, monochorionic twins and PI were not examined in this study. They used RI and compared singleton nomograms, and their previously established twin nomograms in prediction of preeclampsia. They found, using their established twin nomograms, that 14% were screen-positive compared to 3.1% when using nomograms for singletons. Using RI and notching to screen for preeclampsia showed lower positive predictive values when compared to our actual results (29.2% vs. 48%).

It has been shown that in twin pregnancies with uneventful outcomes, sFlt-1 levels, and the sFlt-1/PLGF ratio were increased when compared with singleton pregnancies. No difference could be found in pregnancies with preeclampsia with respect to the sFlt-1/PLGF ratio between twin pregnancies and singleton pregnancies. Overall, significant differences in these parameters between singleton and twin pregnancies were found [25]. This is in line with the difference in mean PI of the uterine arteries and could represent the difference in fetal circulation and placental mass between these two groups.

The second study investigating the relevance of notching and the use of uterine artery PI in prediction of preeclampsia in twin pregnancies did use, in contrast to our study, transvaginal ultrasound [12]. According to our study, they described lower PI values in twin pregnancies compared to singletons, and an association between elevated PI and adverse outcomes related to uteroplacental insufficiency. The use of notching did not add additional power for the detection for any of their adverse outcomes. No association was found for gestational age and mean PI, which is in contrast to most other studies. 

The third study was a retrospective analysis that examined monochorionic and dichorionic twin pregnancies to detect differences between these two groups with respect to uterine PI, notching and fetal and maternal complications [26]. In contrast to the other studies, they found a significant difference in uterine PI between monochorionic an dichorionic twins. Furthermore, the uterine PI was measured later than in our study (22+0 weeks to 24+6 weeks of gestation).

The unique characteristic of our calculations is the differentiation of the uterine PI in the lowest, mean, and highest PI above the 95% centile. This was not used in the other papers, and the use of the highest PI above the 95% centile seems to increase sensitivity for the prediction of preeclampsia. The use of notching increased the sensitivity for preeclampsia further.

In our study, monochorionic and dichorionic twins showed no significant difference regarding the mean, lowest and highest PI of the uterine arteries as well as notching. This is in line with the study of Nicolaides et al., who did not find differences between monochorionic and dichorionic twin pregnancies regarding the PI of the uterine arteries either [27]. Nevertheless, monochorionic twin pregnancies are at a substantially higher risk for developing selective FGR (sFGR), regardless of the occurrence of preeclampsia. The type of sFGR, time of gestational age of diagnosis, and ductus venosus PI identify monochorionic twin pregnancies which are at higher risk for adverse outcome [28].

It would be of high interest and is actually a weakness of the study that we could not include biochemical biomarkers like sFlt-1 and PLGF, and their ratio, together with a uterine artery Doppler for generating predictive models for the development of preeclampsia.

One could criticize that, for the diagnosis of notching, no generally accepted definition is available [29]. However, notching was used in different screening studies and seemed to be consistent between different observers, but final confirmation of this fact is missing [30]. When used for singleton pregnancies, it has been shown that notching increased the screening characteristics significantly in the prediction of preeclampsia. The Doppler waveforms in our study were analyzed by two independent experienced sonographers, and only if both agreed, then notching was used for calculation. 

## 5. Conclusions

Notching increased the screening characteristics for preeclampsia, early preeclampsia, and FGR in twin pregnancies. 

## Figures and Tables

**Table 1:** Patients’ characteristics (Controls vs. Preeclampsia, Controls * vs. Early preeclampsia).

	Controls (*n* = 356)	Preeclampsia (*n* = 24)	Controls * (*n* = 365)	Early Preeclampsia (*n* = 15)
Age, years	31.6 ± 5.7 ^†^	34.0 ± 5.5 ^†^	31.6 ± 5.7	34.1 ± 5.1
BMI, kg/m^2^	24.4 ± 4.4 ^†^	27.0 ± 3.2 ^†^	24.5 ± 4.3 ^†^	27.2 ± 3.5 ^†^
Nulliparity	129 (36)	5 (21)	131 (36)	3 (20)
Smoking	56 (16)	1 (4)	57 (16)	0 (0)
Mode of conception Spontaneous ART	221 (62) ^†^ 135 (38) ^†^	9 (37) ^†^ 15 (63) ^†^	223 (61) 142 (39)	6 (40) 9 (60)
Chorionicity dichorionic twins monochorionic twins	259 (72.8) 97 (27.2)	18 (75) 6 (25)	266 (72.9) 99 (27.1)	11 (73.3) 4 (26.7)

BMI: Body Mass Index, ART: assisted reproductive technology, * Controls: including 9 cases with late preeclampsia, ^†^ *p* < 0.05; Data are mean ± SD or *n* (%).

**Table 2:** Highest, lowest and mean Pulsatility Index above the 95th centile and notching of the uterine artery and relative risk (RR) for preeclampsia.

	Highest PI	Lowest PI	Mean PI	Notching	Notching or Highest PI	Maternal Characteristics	Maternal Characteristics and Notching or Highest PI
Preeclampsia	12.3 (4.7–32.1) *	8.0 (2.8–23.1) *	5.5 (1.8–16.5) ^†^	21.5 (8.4–55.2) *	7 15.6–115.8 *	3.3 (1.1–7.2) ^†^	21.3 (1.6–128.1) ^†^
Early Preeclampsia	22.9 (7.5–70.2) *	5.3 (1.4–20.6) ^†^	7.7 (2.2–26.8) *	25 (8.1–76.8) *	42.4 14.4–308.7 *	1.1 (1.0–1.3)	55.4 (13.1–451.3) ^†^

PI: Pulsatility Index, Data are Relative Risk (95% Confidence Interval), * *p* < 0.001; ^†^ *p* < 0.01.

**Table 3:** Screening characteristics for notching, mean, lowest, highest PI above the 95th centile and combination of notching and the highest PI above the 95th centile.

Characteristic	*N*	Sensitivity	Specificity	PPV	NPV	RR	95% CI
Notching							
Preeclampsia Early Preeclampsia	24 15	12/24 (50%) 9/15 (60%)	344/356 (96%) 344/365 (94%)	50% 30%	96% 98%	21.5 25	8.4–55 8.1–76.8
FGR < 10th centile	78	13/78 (17%)	288/302 (95%)	48%	81%	4.1	1.8-9.1
**Mean PI > 95th centile**							
Preeclampsia Early Preeclampsia	24 15	5/24 (21%) 4/15 (27%)	343/356 (96%) 347/365 (95%)	28% 18%	95% 97%	5.5 7.7	1.8–16.5 2.2–26.8
FGR < 10th centile	78	9/78 (12%)	293/302 (97%)	50%	81%	4.3	1.6–11.2
**Lowest PI > 95th centile**							
Preeclampsia Early Preeclampsia	24 15	6/24 (25%) 3/15 (20%)	343/356 (96%) 347/365 (95%)	32% 18%	95% 97%	8.0 5.3	2.8–23.1 1.4–20.6
FGR < 10th centile	78	7/78 (9%)	293/302 (97%)	44%	81%	3.2	1.2–8.9
**Highest PI > 95th centile**							
Preeclampsia Early Preeclampsia	24 15	11/24 (45%) 8/15 (53%)	343/356 (96%) 347/365 (95%)	46% 31%	96% 98%	12.3 22.9	4.7–32.1 7.5–70.2
FGR < 10th centile	78	11/78 (14%)	289/302 (96%)	46%	81%	3.7	1.6–8.6
**Notching or Highest PI > 95th centile**							
Preeclampsia Early Preeclampsia	24 15	22/24 (91%) 13/15 (87%)	332/356 (93%) 335/365 (91%)	48% 30%	99% 99%	7.0 42.4	15.6–115.8 14.4–308.7
FGR < 10th centile	78	18/78 (23%)	278/302 (92%)	43%	2%	3.2	1.8–6.8

PI: Pulsatility Index; FGR: fetal growth restriction; PPV: positive predictive value; NPV: negative predictive value; RR: relative risk; CI: Confidence Interval.
